# How Noisy Does a Noisy Miner Have to Be? Amplitude Adjustments of Alarm Calls in an Avian Urban ‘Adapter’

**DOI:** 10.1371/journal.pone.0029960

**Published:** 2012-01-04

**Authors:** Hélène Lowry, Alan Lill, Bob B. M. Wong

**Affiliations:** School of Biological Sciences, Monash University, Clayton, Victoria, Australia; University of Lethbridge, Canada

## Abstract

**Background:**

Urban environments generate constant loud noise, which creates a formidable challenge for many animals relying on acoustic communication. Some birds make vocal adjustments that reduce auditory masking by altering, for example, the frequency (kHz) or timing of vocalizations. Another adjustment, well documented for birds under laboratory and natural field conditions, is a noise level-dependent change in sound signal amplitude (the ‘Lombard effect’). To date, however, field research on amplitude adjustments in urban environments has focused exclusively on bird song.

**Methods:**

We investigated amplitude regulation of alarm calls using, as our model, a successful urban ‘adapter’ species, the Noisy miner, *Manorina melanocephala*. We compared several different alarm calls under contrasting noise conditions.

**Results:**

Individuals at noisier locations (arterial roads) alarm called significantly more loudly than those at quieter locations (residential streets). Other mechanisms known to improve sound signal transmission in ‘noise’, namely use of higher perches and in-flight calling, did not differ between site types. Intriguingly, the observed preferential use of different alarm calls by Noisy miners inhabiting arterial roads and residential streets was unlikely to have constituted a vocal modification made in response to sound-masking in the urban environment because the calls involved fell within the main frequency range of background anthropogenic noise.

**Conclusions:**

The results of our study suggest that a species, which has the ability to adjust the amplitude of its signals, might have a ‘natural’ advantage in noisy urban environments.

## Introduction

Animals that rely on acoustic communication must ensure that their vocalizations are not masked by background noise. Individuals may therefore need to modify their signals under different sound conditions if they are to be discernible to conspecifics [1]. Whilst background noise is a ubiquitous feature of natural environments, the level of noise often associated with urban settings represents a particularly formidable challenge for individuals of many species that communicate acoustically [1]–[3]. A common feature of urban environments is constant, loud, anthropogenic noise. Insights gleaned from animals that are reliant on vocal communication, but are nevertheless able to succeed within the challenging acoustic environment posed by cities (described as urban ‘adapters’; [4]), can help us to understand the ability of species to cope with conditions altered by humans [5]–[7].

Some birds in urban environments have the capacity to make vocal adjustments. This is often achieved by altering, for example, the frequency (kHz) [8]–[13] and/or duration [9]–[10] of their signals in such a way as to avoid auditory masking by background noise. Another important form of vocal adjustment, which has been well documented for birds in laboratory studies [14]–[17] but has received less attention under more natural, field conditions [18]–[19], are noise-dependent changes to the amplitude of sound signals.

The ‘Lombard effect’, whereby animals maintain the broadcast area of their vocalizations by increasing vocal amplitude in response to an increase in background noise level [20], has been described for only one urban bird species, the Common nightingale, *Luscinia megarhynchos*. In that species, birds inhabiting noisier locations sang more loudly than those living in quieter locations [21]. Interestingly, a study modeling the benefits of adjustments to vocal amplitude and frequency (kHz) in urban Great tits, *Parus major,* and European blackbirds, *Turdus merula*, suggested that an increase in song amplitude was the more effective means of improving signal transmission in urban noise [22]. However, adjustments to vocal amplitude are energetically costly [23] and some species may simply lack the physiological capacity (e.g. through limitations of the communication system, such as low membrane tension of the trachea or bronchi; [24]) or vocal ‘plasticity’ to make such adjustments [1]. Given that many birds rely heavily on vocalizations to communicate [25], the ability to make amplitude adjustments in noisy environments could have a direct bearing on individual fitness and consequently influence which birds are able to inhabit urban environments.

To date, research on amplitude adjustments in urban birds has focused exclusively on song. Additionally, most studies of urban ‘adapters’ tend to only compare birds in urban and non-urban locations, but we know little about the differences that might occur within the urban environment. Accordingly, we conducted the first wholly urban field study comparing amplitude regulation of the alarm calls of a successful urban ‘adapter’ species. Alarm calls are used to communicate important information among conspecifics (and sometimes among heteorospecifics) on the appropriate response to a perceived threat (i.e. to assemble or flee) [24]. Masking of alarm signals by anthropogenic noise might therefore have a particularly strong impact on a species' success in urban environments.

The Noisy miner is a large (length 26 cm; mass 70–80 g), native, Australian honeyeater (Meliphagidae) currently thriving in noisy, urban environments [26]. The species can be easily identified by its distinct and loud alarm calls [27]. The specific aim of the study was to determine whether Noisy miners call in a manner consistent with the ‘Lombard effect’ in urban environments. We did this by directly comparing the amplitude of their alarm calls in the immediate vicinity of roads with contrasting background noise levels. Additionally, associated behaviours known to improve signal transmission in birds (increases in perching height, use of in-flight calls and call selection; [9], [28], [29]) were investigated as other possible mechanisms employed by Noisy miners that might mitigate the masking effect of urban noise.

## Methods

### Ethical note

From an ethical perspective, dogs are not novel stimuli to the focal species in urban environments, and the behavioural response elicited in Noisy miners is short-lived and appears to have no adverse effects on the birds. Nonetheless, in this project a concerted effort was made to limit disturbance of the birds by visiting each site with the dog only once. Disturbance to birds caused by the dog's presence was also very brief (maximum of 3-min per focal individual) and the dog remained on the lead and was not allowed to approach within 5-m of the perching bird. The dog appeared oblivious to the birds. The study was approved by The Biological Sciences Animal Ethics Committee of Monash University.

### Study sites and locations of bird colonies

Although Noisy miners can breed at any time of year [26], experimental work was conducted in February to April 2010 during the main non-breeding season. Noisy miners were located aurally (by their distinctive alarm calls) and visually whilst walking or driving along arterial roads and residential streets in metropolitan Melbourne, Australia (37°50′S, 145°00′E), where the species is widely distributed. The Noisy miner is a communally breeding species that forms sedentary colonies (home ranges average <250 m in diameter) of varying densities [30]. Therefore a colony was defined as a group of three or more miners at least 500 m away from any other group of conspecifics. Eighty colonies were tested; these were equally divided between arterial road (>5000 vehicles per day) and residential street sites (<500 vehicles per day), which both contained a mixture of native and exotic roadside vegetation.

### Experimental procedure

Recording of adult Noisy miners' calls and observation of associated behaviours were undertaken on weekdays (Mon-Fri) during the peak morning vehicular traffic period (07:00–09:30 hrs; based on Vic Roads Traffic Volume Data [31]), which also coincides with the most vocally-active period of the day (05:00–10:00 hrs) when birds use much of their vocal repertoire [32]. Recordings were only made during dry, still conditions. We recorded alarm calls along a 400 m long transect next to the road. In order to elicit the actual alarm calls, an observer (H.L.) walked the length of the transect at a pace of 0.5 m/s with a domestic dog tethered on a short (1 m) lead. We used a dog to elicit alarm calls because Noisy miners frequently encounter dogs in urban environments and alarm call in response to their presence. On sighting an adult Noisy miner within 5 m of the transect, the observer stopped, instructed the dog to sit beside her, and waited until the sighted individual vocalized. A hand-held Center 322 Data Logger Sound level meter with a 1.3 cm Electret Condenser microphone was positioned so that there was a clear path between the microphone and the vocalizing bird, thus limiting interference from background noise. The time weighting on the meter was set on ‘slow response’ (1 s), the sampling range on ‘auto’ (measuring level range: 30–130 dB) and the frequency on A-weighting (used for general sound-level measurements) for all recordings. The recording continued until the bird had finished vocalizing, which sometimes comprised multiple call bouts. If the bird began vocalizing before the observer and her dog had approached within 5 m, the recording was made from the point where the observer was when the bird began vocalizing. For all recordings, the horizontal distance between the observer and the focal bird's perching location was paced out, and the perching height (m) of the focal bird was measured with a Haglof Electronic clinometer.

**Figure 1:**
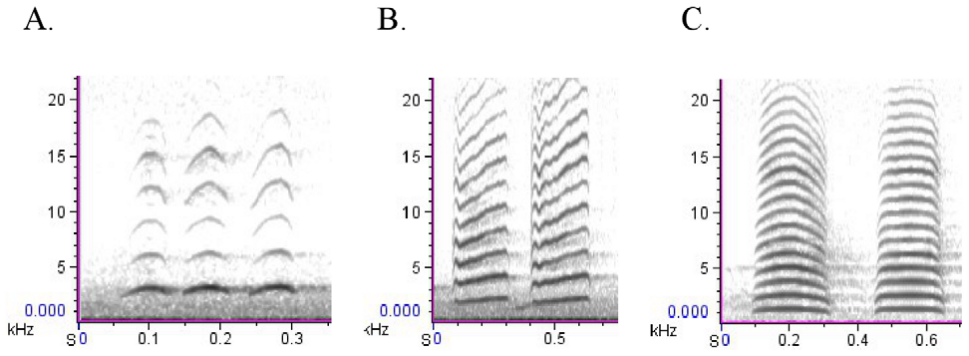
Spectrograms of the three Noisy miner alarm calls. (A) Alarm call (1) is a repeated, single-note, whistle alarm call (associated with aerial predators and mobbing), (B) Alarm call (2) is a loud, repeated, single-note alarm call (associated with ground predators and mobbing), (C) Alarm call (3) is a ‘husky’, repeated, single-note alarm call (also associated with ground predators and mobbing). Call bouts averaged 7 seconds in duration and remained consistent throughout.

During a recording session, the focal bird's call choice (the type of alarm call it employed in response to the dog's presence; see figure 1 for alarm call spectrograms and descriptions), any disturbance other than vehicular traffic within 5 m of the focal bird (e. g. pedestrian walking along footpath) and any other birds present (species, number of individuals and behaviour(s)) within that distance were also noted. The number of conspecifics present within the specified 5 m radius of the focal bird never exceeded 5 adult miners. In instances where miners, other than the focal bird, commenced alarm calling during a recording session, the recording was terminated. Recordings were not conducted if a juvenile Noisy miner was within 5 m of the transect, as adults would be expected to behave differently (i.e. be more aggressive) in the presence of juveniles [26]. If the focal bird did not vocalize within 3 min or flew off, the observer moved on to a ‘new’ individual. If the individual flew off after the observer had made a recording, binoculars were used to it identify where it had gone to ensure that it was not re-sampled. In instances where no call recordings were obtained during an entire site visit, the site was revisited on another day at least 7 days later.

Ambient noise levels were recorded immediately after completion of alarm call recording, using the same equipment and settings. Ambient noise was recorded from the same point as the vocal recording. The observer first directed the hand-held sound-level meter at the bird's perching location and took a 10 s recording, subsequently turning 90^0^ and repeating the process, until a total of four 10 s recordings had been obtained. We also documented, for each site, the number and type of all in-flight calls emitted by any Noisy miners within 10 m either side of the transect line whilst the observer was walking the transect.

### Acoustic analyses

The sound-level meter was directly connected to a PC and recordings were loaded into a data logger spreadsheet using Setup Testlink SE-322 (Sound Level Meter) – RS232 interface software program. For each data set, recording number, start and end time (±1 s), and sound amplitude level (minimum, maximum, and average in decibels (dB)) were noted.

Call recordings were taken at varying distances from the focal bird, so it was necessary to standardize all recordings to a set distance for analysis. Preliminary tests on the effect of distance on sound attenuation showed that there was a significant linear relationship between the amplitude of a call and the ‘actual’ (or direct) distance between the focal bird and the observer. Therefore, we calculated the ‘actual’ distance from the horizontal and vertical distances using Pythagoras' theorem and then converted all recordings to amplitude of the signal at 1 m from the vocalizing bird, as described in Brumm [21]. The signal-to-noise ratio of each recording was subsequently calculated from the standardized data according to the logarithmic computation procedures given in Leader et al. [33].

### Statistics

All data were checked for normality and homogeneity of variances. Two sample t-tests were used to compare call amplitude and corresponding background noise levels for Noisy miners occupying arterial and residential roads, as well as perching height of focal birds in these two locations. An ordinary least squares linear regression was used to identify if there was a significant relationship between call amplitude and the background noise at sites. A Pearson's Chi-squared goodness-of-fit test was used to compare the frequency of in-flight calls between arterial and residential road sites and a Fisher's exact test to examine if there were any significant overall differences in type of alarm call used between Noisy miners occupying the two types of site. Two-way Fisher's exact tests were also employed to determine which particular call types contributed to significant differences between birds inhabiting arterial and residential sites. Unless indicated otherwise, all results are presented as mean±s.e. with alpha set at 0.05. Statistical analyses were conducted with R version 2.20 (The R Foundation for Statistical Computing).

## Results

**Figure 2:**
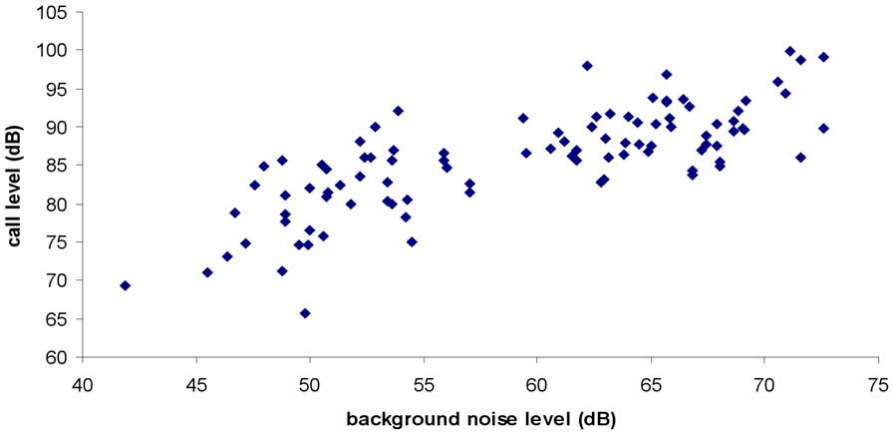
Relationship between environmental background noise level in urban habitat and the amplitude of urban Noisy miner calls. Each datum represents the mean value (dB) for one Noisy miner.

There was a significant difference in background noise level between arterial and residential roads (mean amplitude: arterial = 65.80±0.47dB, residential = 50.83±0.50dB, t = 21.9069, df = 96, p<0.001). Alarm call amplitude of Noisy miners was also greater at arterial than residential roads (mean maximum amplitude (at 1 m distance from bird): arterial = 88.60±0.59dB, residential = 79.53±0.90dB, t = 8.713, df = 95, p<0.001) Overall, a significant relationship was found between the background noise level at a site and the amplitude of Noisy miner alarm calls; individuals at noisier locations called more loudly than those at quieter locations (figure 2; r^2^ = 0.552, df = 95, p<0.001), indicating that Noisy miners were exhibiting the Lombard effect in urban Melbourne. The signal-to-noise ratio was significantly lower on arterial than residential roads (mean: arterial = 2.59±0.07dB, residential = 3.82±0.09dB, t = −11.132, df = 95, p<0.001).

Perching height (mean: arterial = 6.8±0.52m, residential = 6.6±0.73m, t = 0.2147, df = 96, p = 0.831) and use of in-flight calling (χ^2^ = 2.0126, df = 1, p = 0.156) were not significantly different between arterial and residential roads. However, call selection was significantly different between road types (p = 0.03, Fisher's exact test); birds on arterial roads were more frequently heard giving alarm call type 2 (70.4%) than residential road birds (54.6%), whilst the latter used alarm call type 3 (43.2%) more often than arterial road birds (20.4%) (p = 0.04, Fishers exact test). Both these alarm calls are low-frequency in nature (Lowry et al. unpublished data), and so would not be expected to avoid masking by background noise. The whistle alarm call (alarm call 1), which would elude masking by low-frequency anthropogenic noise, was not significantly different in occurrence between road types. It was only recorded on 5 occasions on arterial roads and once on residential roads (p = 0.23, Fisher's exact test).

## Discussion

### Amplitude adjustments

Overall, our results indicate that Noisy miners at noisier locations (busier arterial roads) were calling more loudly than individuals at quieter locations (residential streets). A similar pattern of amplitude adjustments in relation to background noise levels (the ‘Lombard effect’) has been shown in the song of another urban ‘adapter’, the Common nightingale [21]. By increasing the amplitude of the signal, and thus increasing the signal-to-noise ratio (hereafter SNR), the ‘active space’ of the signal is maintained, so that conspecifics can detect a vocalization in noise over a larger area [34]. The lower SNR recorded at noisier sites suggests that the magnitude of the amplitude increase by Noisy miners on arterial roads is not enough to prevent masking of signals from background noise. However, if miners are communicating over small enough distances, the active space required to be heard by conspecifics might be relatively small. Birds vary in their ability to hear in noise and thus the required SNR for an individual to communicate successfully with a conspecific in noise will differ among species [35]. There is some suggestion that passerines have poorer auditory sensitivity at lower sound frequencies [36], which correspond with the main frequency range of anthropogenic noise (see [37]).

There are numerous laboratory-based studies demonstrating the ‘Lombard effect’ for animal ‘calls’ (e.g. Budgerigar, *Melopsittacus undulatus* [14]; Common marmoset, *Callithrix jacchus* [38]; Domestic fowl, *Gallus gallus domesticus* [39]). Amplitude adjustments of calls have also being demonstrated in ‘natural’ environments for a single bird (Blue-throated hummingbird, *Lampornis clemenciae* [19]) and frog species (leptodactylid frog, *Eupsophus calcaratus* [18]). Interestingly, a recent study by Nemeth and Brumm [22] suggests that amplitude adjustments are a more effective means of reducing sound attenuation in noisy conditions than frequency (kHz) adjustments (see also [8], [9], [10], [12], [40], [41] for examples of frequency adjustments in songbirds). This may help to explain why some bird species whose vocal signals fall within the main frequency range of anthropogenic noise (described as 1–2 kHz; [37]) and which lack the vocal flexibility to make frequency (kHz) adjustments (calls are usually innate and are therefore expected to be less ‘flexible’ than learnt vocalizations such as song; [42]), are able to inhabit noisy urban environments. This also highlights the importance of looking at bird ‘calls’ (as apposed to song) given birds that call are more likely to need to employ temporal vocal mechanisms that are more ‘plastic’, such as amplitude adjustments in noisy conditions.

In the current study, signal amplitudes exceeded 90 dB in some instances, demonstrating that Noisy miners have the vocal capacity to easily exceed the background sound amplitude averages recorded in this (see section ‘Results’) and other studies measuring urban noise (see [13], [21]). Surprisingly, high-amplitude signal production is not limited to larger birds; Brackenbury [43] measured amplitudes ranging from 74–100 dB (at 1 m from vocalizing bird) in 17 European songbird species, some of which were <20 g in body weight. However, high-amplitude vocal output is energetically costly (i.e. involves an increased rate of oxygen consumption) [24] and, consequently, would be more costly to a small than a large bird, due to its higher mass-specific metabolic rate [24]. This is reflected in Brackenbury's [43] study, which found that generally the poorest performances (lower maximum total sound power) came from the smaller birds. Thus, smaller birds may experience particular difficulty in communicating vocally under continuously noisy conditions such as those encountered in cities. The ability to produce high-amplitude vocal signals over extended periods is likely to be an important pre-requisite for birds to successfully colonize noisy, urban environments, and a comparison of the capacity to do this in other urban ‘adapters’ and ‘avoiders’ (*sensu* [5]) would be an interesting extension of the present study.

### In-flight calls and perching height

Perching height adjustment and in-flight calling are both indirect mechanisms that can be employed by birds to improve signal broadcasting. By increasing its elevation, a bird can create a clearer transmission pathway for its signal (i.e. ground attenuation and wind and temperature ‘shadow zones’ have a greater impact at lower elevations *sensu* [44]) and thus improve its SNR [45], whilst use of in-flight calling allows the signaler to increase its vocal range to receivers on the ground [44]. This has been demonstrated for the European blackbird [46] and Green hylia, *Hylia praxina* [28]. However, in the current study, we found that Noisy miners showed no difference in perching height or the frequency of in-flight calls between arterial and residential roads. The similar average perching heights of vocalizing Noisy miners in both road types may reflect similarities in the roadside vegetation (e.g. in tree height).

### Call selection

Recently Luther and Baptister [29] found that White-crowned sparrows, *Zonotrichia leucophry*, favoured songs with the highest minimum frequencies over lower frequency songs in urban environments. We also found significant differences in alarm call selection between Noisy miners inhabiting arterial and residential roads. However, the two alarm calls whose use differed between road types for the current study had low frequencies (<2 kHz) (Lowry et al. unpublished data) that were within the frequency range (1–2 kHz) of background anthropogenic noise (see [37]). Therefore use of either of these alarm calls would be unlikely to prevent masking by background noise in urban habitats. Notably, it is the peak frequency that is most critical in determining the active space of a signal, and all three Noisy miner alarm calls have peak frequencies above the main frequency range of urban noise (Lowry et al. unpublished data). Theoretically this difference in alarm call selection could simply reflect a difference in response to the approaching observer during recording sessions; Noisy miners at higher-disturbance sites (arterial roads) might experience pedestrians walking dogs less often than birds on residential roads and thus perceive the threat differently and select a different alarm call for that reason. However, research on Noisy miner alarm calling has shown that these birds can alternate between all three of the described calls in response to the same threat, making the observed differences in the current study difficult to interpret [27].

### Conclusions

Urban Noisy miners appear to exhibit the ‘Lombard effect’ in avoiding masking of important vocal signals in noisy urban environments by amplitude adjustment. However, there was no evidence to suggest that other behavioural mechanisms known to improve signal transmission in ‘noise’ in birds, such as increases in perching height and in-flight calling were being employed more commonly in noisier areas. Whilst we found significant differences in alarm call selection between Noisy miners inhabiting arterial and residential roads, the low frequencies (kHz) of the calls selected fell within the main frequency range of anthropogenic urban noise (1–2 kHz) and thus use of these calls is unlikely to constitute a vocal modification made in response to sound-masking in the urban environment. Our findings, in conjunction with other research on signal amplitude adjustments in birds, seem to suggest that the type (i.e. call or song) and the frequency (kHz) of the signals used may not necessarily limit a species' capacity to mitigate vocal masking by urban noise. A species, which has the ability to adjust the amplitude of its signals, might have a ‘natural’ advantage in noisy urban environments.
